# Correction: Reis et al. Recombinant Human Peptide Growth Factors, Bone Morphogenetic Protein-7 (rhBMP7), and Platelet-Derived Growth Factor-BB (rhPDGF-BB) for Osteoporosis Treatment in an Oophorectomized Rat Model. *Biomolecules* 2024, *14*, 317

**DOI:** 10.3390/biom16071041

**Published:** 2026-07-17

**Authors:** Thamara Gonçalves Reis, Alice Marcela Sampaio Del Colletto, Luiz Augusto Santana Silva, Bruna Andrade Aguiar Koga, Mari Cleide Sogayar, Ana Claudia Oliveira Carreira

**Affiliations:** 1 Cell and Molecular Therapy Group (NUCEL), School of Medicine, University of São Paulo, São Paulo 01246-903, SP, Brazil; thamara.gr23@gmail.com (T.G.R.); alicesampaiodelcolletto@gmail.com (A.M.S.D.C.); brunaaguiar5@gmail.com (B.A.A.K.); 2 Biotechnology Graduate Program (PPG-USP), University of São Paulo, São Paulo 05508-900, SP, Brazil; 3 Surgery Department, School of Veterinary Medicine and Animal Science, University of São Paulo, São Paulo 05508-270, SP, Brazil; 4 PathoDxVet, São Paulo 02925-000, SP, Brazil; lzs1.augusto@gmail.com; 5 Biochemistry Department, Chemistry Institute, University of São Paulo, São Paulo 05588-000, SP, Brazil

In the original publication [1], there was a mistake in Figures 6 and 7 as published. Due to an inadvertent error during the final assembly of the figures, the images for the PDGF-BB 2×/week group in these figures were incorrectly replaced by duplicates from another experimental group. The corrected Figure 6 and Figure 7 appear below. The authors state that the scientific conclusions are unaffected. This correction was approved by the Academic Editor. The original publication has also been updated.

## Figures and Tables

**Figure 6:**
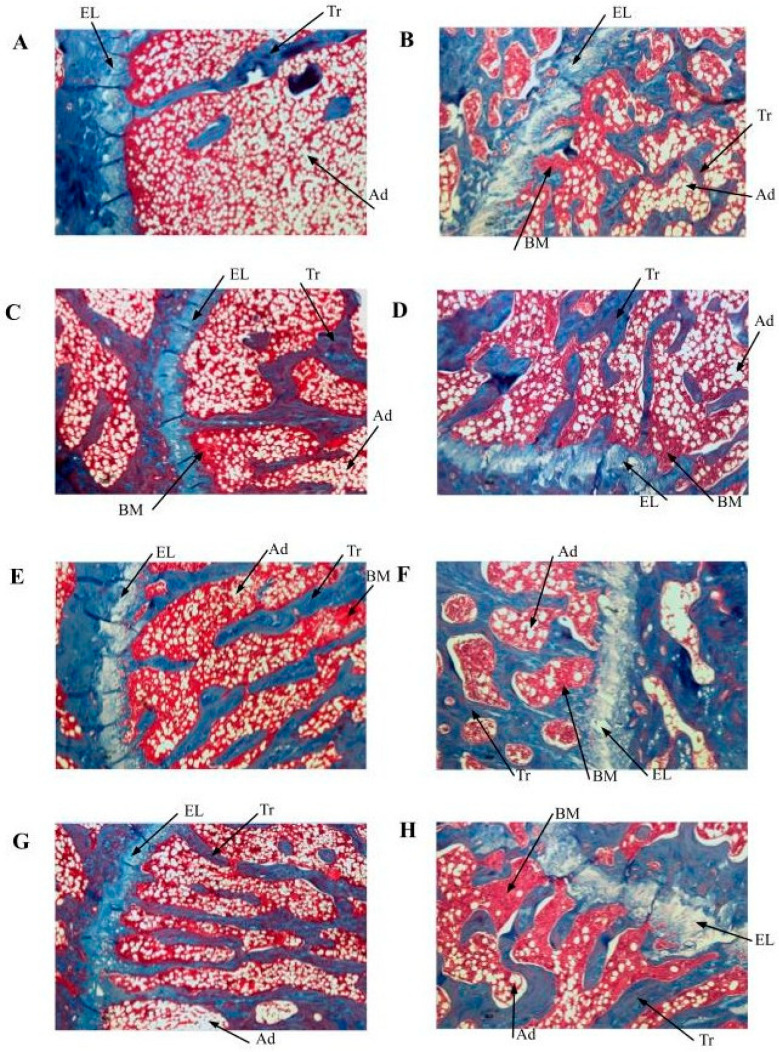
Masson’s trichrome stained histological slides comparing the different treatments. (**A**): Vehicle; (**B**): Zoledronic acid; (**C**): PDGF-BB 2×/week; (**D**): PDGF-BB 1×/week; (**E**): BMP-7 2×/week (**F**): BMP-7 1×/week; (**G**): PDGF-BB + BMP-7 2×/week (**H**): PDGF-BB + BMP-7 1×/week. Scale bar: 100 µm. In blue are collagen fibers, cytoplasm in red, and cell nuclei in purple. Ad: Adipocytes; BM: Bone marrow; Tr: Trabeculae; EL: Epiphyseal line.

**Figure 7:**
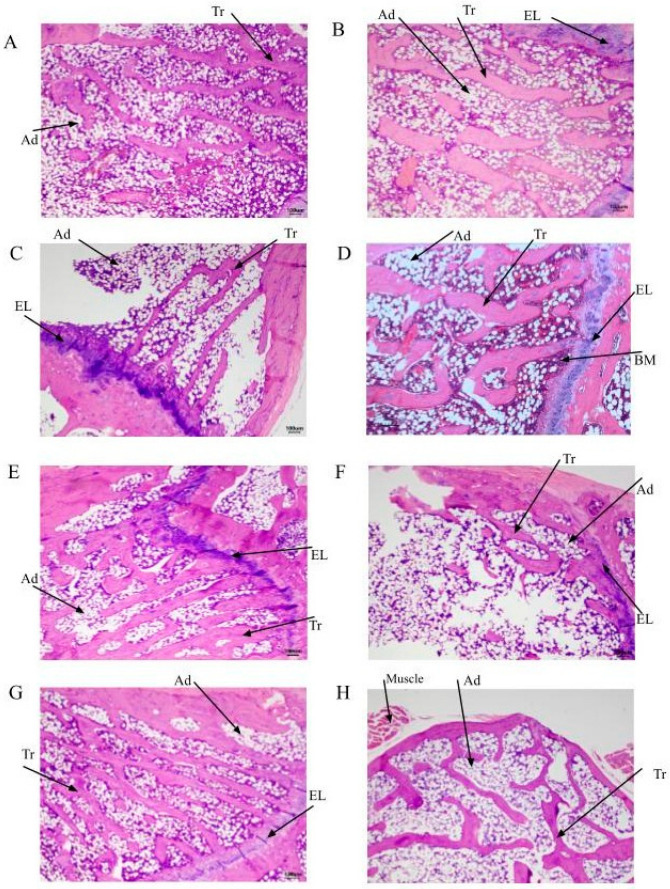
Histological slides stained with HE comparing the different groups analyzed. (**A**): Vehicle; (**B**): Zoledronic acid; (**C**): PDGF-BB 2×/week; (**D**): PDGF-BB 1×/week; (**E**): BMP-7 2×/week (**F**): BMP-7 1×/week; (**G**): PDGF-BB + BMP-7 2×/week (**H**): PDGF-BB + BMP-7 1×/week. Scale bar: 100 µm. In pink/reddish are the cytoplasm and collagen fibers, in purple were stained the cell nuclei. Ad: Adipocytes; BM: Bone marrow; Tr: Trabeculae; EL: Epiphyseal line.
